# Development of a Cost-Effective Method for Capripoxvirus Genotyping Using Snapback Primer and dsDNA Intercalating Dye

**DOI:** 10.1371/journal.pone.0075971

**Published:** 2013-10-07

**Authors:** Esayas Gelaye, Charles Euloge Lamien, Roland Silber, Eeva S. M. Tuppurainen, Reingard Grabherr, Adama Diallo

**Affiliations:** 1 Animal Production and Health Laboratory, Joint FAO/IAEA Division of Nuclear Techniques in Food and Agriculture, Department of Nuclear Sciences and Applications, International Atomic Energy Agency, Vienna, Austria; 2 Institute for Veterinary Disease Control, Austrian Agency for Health and Food Safety, Moedling, Austria; 3 Capripoxvirus Reference Laboratory, The Pirbright Institute, Pirbright, Woking, Surrey, United Kingdom; 4 Institute of Applied Microbiology, University of Natural Resources and Life Sciences, Vienna, Austria; 5 Institute of Applied Genetics and Cell Biology, University of Natural Resources and Life Sciences, Vienna, Austria; 6 Research and Diagnostic Laboratories, National Veterinary Institute, Debre Zeit, Ethiopia; Institut National de la Santé et de la Recherche Médicale U 872, France

## Abstract

Sheep pox virus (SPPV), goat pox virus (GTPV) and lumpy skin disease virus (LSDV) are very closely related viruses of the *Capripoxvirus* (CaPV) genus of the *Poxviridae* family. They are responsible for sheep pox, goat pox and lumpy skin disease which affect sheep, goat and cattle, respectively. The epidemiology of capripox diseases is complex, as some CaPVs are not strictly host-specific. Additionally, the three forms of the disease co-exist in many sub-Saharan countries which complicates the identification of the virus responsible for an outbreak. Genotyping of CaPVs using a low-cost, rapid, highly specific, and easy to perform method allows a swift and accurate identification of the causative agent and significantly assists in selecting appropriate control and eradication measures, such as the most suitable vaccine against the virus during the outbreaks. The objective of this paper is to describe the design and analytical performances of a new molecular assay for CaPV genotyping using unlabelled snapback primers in the presence of dsDNA intercalating EvaGreen dye. This assay was able to simultaneously detect and genotype CaPVs in 63 samples with a sensitivity and specificity of 100%. The genotyping was achieved by observing the melting temperature of snapback stems of the hairpins and those of the full-length amplicons, respectively. Fourteen CaPVs were genotyped as SPPVs, 25 as GTPVs and 24 as LSDVs. The method is highly pathogen specific and cross platform compatible. It is also cost effective as it does not use fluorescently labelled probes, nor require high-resolution melting curve analysis software. Thus it can be easily performed in diagnostic and research laboratories with limited resources. This genotyping method will contribute significantly to the early detection and genotyping of CaPV infection and to epidemiological studies.

## Introduction

Capripox is a severe disease of sheep, goat and cattle characterized by fever, lymphadenopathy, generalized papules, nodules or vesicles in the skin, internal pox lesions particularly in the lungs, and death [1]. Sheep pox virus (SPPV), goat pox virus (GTPV) and lumpy skin disease virus (LSDV) belong to the genus *Capripoxvirus* (CaPV) within the *Poxviridae* family [2]. According to the affected host, sheep, goat or cattle, the disease is named sheep pox (SPP), goat pox (GTP) or lumpy skin disease (LSD). These diseases are categorized as notifiable diseases by the World Organization for Animal Health (OIE) [3].

Capripox affects the ruminant production systems in Africa, the Middle East and Asia. The existence of SPP and GTP in Turkey and Greece and LSD in Israel and Lebanon raises concerns that capripox diseases will become a threat to European countries as well [2], [4]. The economic impact of SPP, GTP and LSD is substantial due to significant production losses, decreased quality of skin and hides, restricted access to the global trade of live animals and animal products, and increased costs of the control and eradication measures [4].

The geographical distribution of LSD differs from that of SPP and GTP which are endemic in Africa, north of the Equator, Asia, the Middle East and some southern European countries [5]–[9]. Lumpy skin disease is currently endemic in most African countries and in the Middle East [4], [10]–[12]. With the exception of southern African countries, all three capripox diseases co-exist in sub-Saharan Africa creating a serious challenge for the identification of the circulating viral genotype during the outbreak.

CaPVs are mainly classified using the name of the host-species from which the virus was originally isolated although it has been reported that some strains are not host-specific [13]–[15]. Recent molecular based studies have shown that CaPVs are phylogenetically and genetically distinct giving a basis for the molecular differentiation of CaPVs [13]–[17].

For many years, CaPV genotyping has been based on the electrophoretic patterns of viral genome isolates following digestion with restriction enzymes [18]. This method is time-consuming and requires large amount of viral material, therefore cannot be applied in a routine basis. More recently, gene sequencing has been proposed for CaPV genotyping [13], [15], [19]; however, this can only be applied for selected samples owing to its cost. The only rapid method available so far for a routine genotyping of the three CaPVs is a real-time PCR assay based on dual hybridization probe technology [14]. However, the use of this method for virus detection and genotyping is costly since it requires the use of two fluorescently labelled probes and specialized real-time PCR machines which accommodate the FRET technology.

Thus CaPV genotyping using a cost-effective, rapid, highly sensitive and specific, and easy to perform method is urgently needed by diagnostic laboratories in countries endemic for LSD, SPP and GTP. The accurate and rapid identification of the virus will assist in the appropriate vaccine selection and will improve the prospects for the control and eradication of the disease.

High-Resolution melting of small sized PCR products in the presence of saturating DNA dye, such as LCGreen and EvaGreen, offers means to develop cost-effective genotyping assays in which, unlabelled probes can be added to increase the specificity [20]–[23].

We investigated the possibility of using an unlabelled probe strategy based on a snapback primer and the intercalating dsDNA EvaGreen dye [24]–[26] for CaPV genotyping using the fluorescent melting curve analysis of the PCR products. Snapback primers are oligonucleotides that include as a probe element a 5′-tail that is complementary to the extension product of the primer and creating a hairpin loop in the single-stranded product [26]. In the present paper, we report on the design and analytical performances of this newly developed molecular assay for genotyping of CaPVs.

## Materials and Methods

### Ethics Statement

Swab samples collected from animals that were infected experimentally with capripoxviruses were used in the present study. The animal experiment was undertaken at the Laboratoire Central Vétérinaire (LCV) of Mali in 2009 and the results are being considered for publication elsewhere. This research laboratory in Mali did not have an operational animal ethics committee at the time of the experimental work. Nevertheless, it has received the necessary authorization to conduct the study from the Ministry of Livestock and Fisheries in Mali. In addition, all experiments were carried out according to the guidelines in the Guide to the Care and Use of Experimental Animals provided by the French Ministry of Agriculture.

None of the authors in this paper participated directly in this animal experiment.

Virulent field isolates and clinical specimens collected from outbreaks at various geographical locations were also used in this study. Skin lesions collected from capripox suspected animals in South Africa, Sudan and Ethiopia were received for viral diagnosis at the Onderstepoort Veterinary Institute, South Africa and the National Veterinary Institute of Ethiopia for viral diagnosis. DNA extracts from skin lesions were forwarded to the authors’ laboratory for further characterization. Additionally, skin lesions collected from capripox suspected animals received at the Central Veterinary Laboratory of Kenya for diagnosis, were forwarded to the authors’ laboratory for capripoxvirus genotype confirmation. Viral isolates including non-capripoxviruses were also used in this study. All pathological samples and viral isolates were handled in the biosafety level 3 containment of the Institute for Veterinary Disease Control, Austria.

### Target Gene and Primers Design

**Figure 1:**
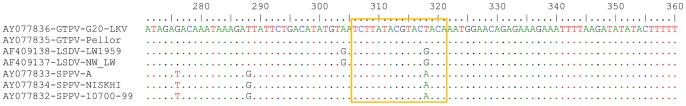
Nucleotides sequence alignment of the RPO30 gene of CaPVs highlighting the snapback tail binding site. The RPO30 gene sequences of 7 CaPVs representing GTPVs, LSDVs and SPPVs were aligned. A sequence of sixteen nucleotides complementary to the snapback tail in GTPV (100% match), as well as the corresponding positions in SPPV and LSDV are shown in the box. Note the targeted single nucleotide mismatches inside the box: T:A between GTPV and SPPV, and T:G between GTPV and LSDV. Conserved nucleotides are shown as dots.

Snapback and reverse primers were designed to target the 30 kDa RNA polymerase subunit (RPO30) gene of CaPVs [13], [17], [27]. Primers were designed to allow the amplification, in all CaPVs, of a 96 base pair (bp) fragment corresponding to position 27875 to 27970 of the SPPV A genome (AY077833). These primers were selected using AlleleID® version 6 software (Premier Biosoft International, Palo Alto, CA, USA). Within this amplicon, a snapback tail of 16 bases length was designed manually to match 100% with GTPV and presented a T:A mismatch with SPPV and a T:G mismatch with LSDV (Figure 1). This snapback tail, whose sequence is complementary to the extension product of the forward primer, was added to the 5′ end of the forward primer. Two nucleotides (GG) not relevant to the extension product of the forward primer were added to the 5′ end of the snapback tail (Table 1) to prevent it from being extended once it anneals to its complementary sequence. The snapback primer serves as both primer and probe. All primers were synthesized by VBC Biotech, Austria and purified by reverse-phase high-performance liquid chromatography. The specificity of the primers sequence was checked by using the Basic Local Alignment Search Tool (NCBI/Primer-BLAST, http://blast.ncbi.nlm.nih.gov./Blast.cgi). The secondary structures of the primers and the anticipated PCR amplicons were studied using the DNA folding form of the Mfold Web Server (http://mfold.rna.albany.edu/?q=mfold) [28].

**Table 1:** Sequences of the snapback and reverse primers.

Primers	Sequences
Snapback	5′-ggTGT**A**GTACGTATAAGATTATCGTATAGAAACAAGCCTTTA-3′
Reverse	5′-AATTTCTTTCTCTGTTCCATTTG-3′

The snapback tail of 16 bases is shown as underlined; 2 nucleotides mismatch at the 5′end are indicated as lowercase; and mismatch nucleotide as underlined bold (A).

### Samples and Viral DNA Extraction

Details of 63 CaPV positive samples and 5 non-capripoxvirus samples used in this study are listed in Table 2. These consisted of cell-culture adapted vaccine strains (n = 4), virulent field isolates (n = 27) and clinical specimens (n = 32) collected from outbreaks at various geographical locations. Additionally, swab samples from experimentally infected animals were included. For tissue samples, 10% w/v homogenate was prepared in sterile phosphate buffer saline (PBS). Swabs were re-suspended in 1 ml PBS. A volume of 200 µL of pathological sample suspension or infected cell culture supernatant was mixed with 800 µL RLT Plus lysis buffer (Qiagen, Germany). Viral DNA was extracted using a commercial AllPrep DNA/RNA extraction kit (Qiagen, Germany) following the manufacturer’s instructions.

**Table 2:** Capripoxviruses and other viruses tested by the snapback primer genotyping assay.

No	Strain name	Origin	Source	Viral DNAextracted from	Speciesorigin	Genotyping result
1	SPPV Turkey/98 Van 2	Turkey	VCRI-Pendik/Turkey	Cell culture	Sheep	SPPV
2	SPPV Turkey/98 Sivas	Turkey	VCRI-Pendik/Turkey	Cell culture	Sheep	SPPV
3	SPPV Turkey/98 Denizli	Turkey	VCRI-Pendik/Turkey	Cell culture	Sheep	SPPV
4	SPPV Turkey/98 Çorum	Turkey	VCRI-Pendik/Turkey	Cell culture	Sheep	SPPV
5	SPPV Turkey/98 Darica	Turkey	VCRI-Pendik/Turkey	Cell culture	Sheep	SPPV
6	GTPV Turkey/98 Denizli	Turkey	VCRI-Pendik/Turkey	Cell culture	Goat	GTPV
7	SPPV Algerie/93 Djelfa	Algeria	INMV-LCV/Algeria	Cell culture	Sheep	SPPV
8	SPPV Algerie/05 Illizi	Algeria	INMV-LCV/Algeria	Cell culture	Sheep	SPPV
9	GTPV Ghana	Ghana	IAH-Pirbright/UK	Cell culture	Goat	GTPV
10	GTPV Bangladesh/86	Bangladesh	IAH-Pirbright/UK	Cell culture	Goat	GTPV
11	GTPV Oman/84	Oman	IAH-Pirbright/UK	Cell culture	Goat	GTPV
12	GTPV India/83	India	IAH-Pirbright/UK	Cell culture	Goat	GTPV
13	GTPV Iraq/61 Gorgan	Iraq	IAH-Pirbright/UK	Cell culture	Goat	GTPV
14	GTPV Yemen/83	Yemen	IAH-Pirbright/UK	Cell culture	Goat	GTPV
15	SPPV Nigeria C4	Nigeria	IAH-Pirbright/UK	Cell culture	Sheep	SPPV
16	**GTPV Saudi Arabia/93**	Saudi Arabia	IAH-Pirbright/UK	Cell culture	**Goat**	**SPPV**
17	**SPPV OMAN/84**	Oman	IAH-Pirbright/UK	Cell culture	**Sheep**	**GTPV**
18	**SPPV KS-1**	Kenya	HSL-AGES/Austria	Cell culture	**Sheep**	**LSDV**
19	LSDV Egypt/89 Ismalia	Egypt	HSL-AGES/Austria	Cell culture	Cattle	LSDV
20	SPPV Morocco vaccine	Morocco	Biopharma/Morocco	Cell culture	Sheep	SPPV
21	LSDV Sudan 99 Atbara	Sudan	CVRL/Sudan	Skin lesion	Cattle	LSDV
22	LSDV RSA 06 Springbok	South Africa	OVI/South Africa	Skin lesion	Springbok	LSDV
23	LSDV RSA 08 M143/08 10/6/08	South Africa	OVI/South Africa	Skin lesion	Cattle	LSDV
24	LSDV RSA/07 Brahman	South Africa	OVI/South Africa	Skin lesion	Cattle	LSDV
25	LSDV RSA/00 OP126402	South Africa	OVI/South Africa	Skin lesion	Springbok	LSDV
26	LSDV RSA06 D.19353	South Africa	OVI/South Africa	Skin lesion	Cattle	LSDV
27	LSDV RSA/54 Haden	South Africa	OVI/South Africa	Cell culture	Cattle	LSDV
28	GTPV Desse I	Unknown	CIRAD/France	Cell culture	Goat	GTPV
29	**GTPV Nigeria goat vaccine**	Nigeria	CIRAD/France	Cell culture	**Goat**	**SPPV**
30	LSDV Burkina Banfora	Burkina Faso	CIRAD/France	Cell culture	Cattle	LSDV
31	SPPV Sangalcam/88	Senegal	CIRAD/France	Cell culture	Sheep	SPPV
32	LSDV Niger Tougounous	Niger	CIRAD/France	Cell culture	Cattle	LSDV
33	GTPV Burkina Benogo 3 A	Burkina Faso	CIRAD/France	Cell culture	Goat	GTPV
34	GTPV Chad VC6	Chad	CIRAD/France	Cell culture	Goat	GTPV
35	GTPV Chad VC8	Chad	CIRAD/France	Cell culture	Goat	GTPV
36	SPPV vaccine Nigeria/99- 77	Nigeria	CIRAD/France	Cell culture	Sheep	SPPV
37	SPPV Niger/88	Niger	CIRAD/France	Cell culture	Sheep	SPPV
38	Embu/B338/2011	Kenya	CVL/Kenya	Nodule	Cattle	LSDV
39	Marsabit/B291/2007	Kenya	CVL/Kenya	Nodule	Cattle	LSDV
40	Kiambu/G143/2009	Kenya	CVL/Kenya	Skin scrapping	Goat	GTPV
41	**Kitengela/O58/2011**	Kenya	CVL/Kenya	Skin scrapping	**Sheep**	**GTPV**
42	**Kitengela/O59/2011**	Kenya	CVL/Kenya	Skin scrapping	**Sheep**	**GTPV**
43	Bungoma/B624/2010	Kenya	CVL/Kenya	Skin scrapping	Cattle	LSDV
44	Fairfield/B01/2009	Ethiopia	NVI/Ethiopia	Skin lesion	Cattle	LSDV
45	Kajima/B01/2009	Ethiopia	NVI/Ethiopia	Skin lesion	Cattle	LSDV
46	EIAR/B01/2009	Ethiopia	NVI/Ethiopia	Skin lesion	Cattle	LSDV
47	**Akaki/O01/2008**	Ethiopia	NVI/Ethiopia	Skin lesion	**Sheep**	**GTPV**
48	NVI/G01/2009	Ethiopia	NVI/Ethiopia	Skin lesion	Goat	GTPV
49	Assosa/G01/2010	Ethiopia	NVI/Ethiopia	Skin lesion	Goat	GTPV
50	**Metekel/O01/2010**	Ethiopia	NVI/Ethiopia	Skin lesion	**Sheep**	**GTPV**
51	Adama/B01/2011	Ethiopia	NVI/Ethiopia	Skin lesion	Cattle	LSDV
52	Adama/B02/2011	Ethiopia	NVI/Ethiopia	Skin lesion	Cattle	LSDV
53	Mojo/B01/2011	Ethiopia	NVI/Ethiopia	Skin lesion	Cattle	LSDV
54	Mojo/B02/22011	Ethiopia	NVI/Ethiopia	Skin lesion	Cattle	LSDV
55	Wenji/B01/2011	Ethiopia	NVI/Ethiopia	Skin lesion	Cattle	LSDV
56	Wenji/B02/2011	Ethiopia	NVI/Ethiopia	Skin lesion	Cattle	LSDV
57	Wenji/B03/2011	Ethiopia	NVI/Ethiopia	Skin lesion	Cattle	LSDV
58	Chagni/G01/2012	Ethiopia	NVI/Ethiopia	Skin lesion	Goat	GTPV
59	Chagni/G02/2012	Ethiopia	NVI/Ethiopia	Skin lesion	Goat	GTPV
60	Chagni/G03/2012	Ethiopia	NVI/Ethiopia	Skin lesion	Goat	GTPV
61	Chagni/G04/2012	Ethiopia	NVI/Ethiopia	Skin lesion	Goat	GTPV
62	Chagni/G05/2012	Ethiopia	NVI/Ethiopia	Skin lesion	Goat	GTPV
63	**Chagni/O06/2012**	Ethiopia	NVI/Ethiopia	Skin lesion	**Sheep**	**GTPV**
	**Non Capripoxviruses**					
1	Orf virus D1701	Germany	HSL-AGES/Austria	Cell culture	Sheep	Neg
2	Orf virus CE030ODV	unknown	HSL-AGES/Austria	Cell culture	Sheep	Neg
3	Orf virus Debrezeit/2012	Ethiopia	NVI/Ethiopia	Skin lesion	Sheep	Neg
4	Bovine Papular Stomatitis virus M1	Germany	HSL-AGES/Austria	Cell culture	Cattle	Neg
5	PPRV Nigeria 75-1	Nigeria	CIRAD/France	Cell culture	goat	Neg

The strains genotyped outside the group corresponding to the name of their host of origin are marked in bold.

Abbreviations: CIRAD = Centre de Coopération Internationale en Recherche Agronomique pour le Dévelopement; CVL: Central Veterinary Laboratories; CVRL = Central Veterinary Research Laboratories Centre; HSL-AGES = High Security Laboratory, Austrian Agency for Health and Food Safety; IAH = Institute for Animal Health; INMV-LCV = Institut National de la Médecine Vétérinaire, Laboratoire Central Vétérinaire; NVI = National Veterinary Institute; OVI = Onderstepoort Veterinary Institute; VCRI = Veterinary Control and Research Institute.

### Positive Controls

Plasmids containing the full RPO30 gene sequence of GTPV Denizli, SPPV Denizli and LSDV Ismalia described by Lamien et al. [13], and available in the laboratory were used. The concentration of the plasmids was determined following the steps described by Lamien et al. [13]. The plasmid of each virus genotype was serially diluted in 10-fold starting from 10^8^ copies using herring sperm DNA (5 ng/µL) and kept at −20°C until analysis.

### PCR and Melting Curve Acquisition

The PCR was set up in a 20 µL reaction volume containing 500 nM snapback primer, 40 nM reverse primer, 1x SsoFast EvaGreen Supermix (BioRad) and sample DNA. PCR was performed in a CFX96™ real-time PCR detection system (Bio-Rad Laboratories, Inc.) with an initial denaturation step at 95°C for 3 min, followed by 45cycles of 95°C for 15sec and 58°C for 80sec using a Low Profile Hard-Shell® 96-well PCR plate (Bio-Rad). The product was then denatured at 95°C (held for 1 min), cooled to 40°C (held for 1 min), and heated continuously at 0.5°C/10sec with fluorescence acquisition from 40°C to 85°C. Each sample was tested in duplicate and every PCR run included no-template and positive GTPV, LSDV and SPPV controls. The melting temperatures were analysed using the CFX™ Manager software version 2.0 (Bio-Rad) and the correspondent curves were displayed as negative first-derivative plots of fluorescence with respect to temperature. High-Resolution Melting (HRM) analysis, a post-PCR melting analysis method used to identify variations in nucleic acid sequences, was also used to plot the melting profile of the three genotypes using the Precision Melt Analysis™ software (Bio-Rad). Normalized melt curves and difference in curves were acquired by selecting pre-and post-melt regions for snapback hairpin and full-length amplicons separately.

Alternatively, the current method was tested using different PCR machines to evaluate the possibility of performing the assay using the available PCR platforms. Thus, PCR was performed on a conventional PCR machine (C1000, Bio-Rad) and the PCR product transferred to the CFX96 real-time PCR machine (Bio-Rad) for melting acquisition. Likewise, both PCR and melting analysis steps were conducted in the 7500 Fast Real-Time PCR System (Life Technologies), the LightCycler® 480 Real-Time PCR System (Roche), the Rotor-Gene Q (Qiagen) and the MiniOpticon™ Real-Time PCR Detection System (BioRad), in addition to the CFX96 ™ Real-Time PCR Detection System (BioRad).

### Limit of Detection for Each Genotype

The analytical sensitivity of the method was assessed by amplifying five different concentrations (200, 160, 120, 80 and 40 copies/reaction) of each plasmid containing GTPV Denizli, SPPV Denizli and LSDV Ismalia RPO30 gene. The limit of detection (LOD) of each genotype was evaluated by testing the diluted plasmids in pentaplicate separately at different days on five separate occasions. The data from each PCR reaction was recorded and subjected to probit regression analysis using the STATGRAPHICS Centurion XV Version 15.2.12 software package (StatPoint Technologies, Warrenton, VA, USA).

Because this method aims simultaneously detecting and genotyping the CaPVs, the criteria for a PCR to be considered as positive for the LOD determination were taking into account both amplification plot and the shape of the melting peak of the snapback stem. Only when the melting curve allowed univocal identification of the genotype, in addition to the positive amplification plot, the reaction was considered as positive for LOD determination. For samples that showed positive amplifications but could not be genotyped due to low initial copy number, the number of cycles in the PCR was increased from 45 to 50 cycles to allow the accumulation of more single stranded DNA for the hybridization of the snapback tail.

### Discriminating Power of the Assay

The sensitivity and specificity of the assay was tested by comparing the amplification and genotyping level of CaPV DNA samples extracted from infected cell culture and pathological samples of skin lesions collected from different geographical regions (Table 2) and swabs collected from animals that were infected experimentally. Moreover, the analytical specificity of the assay was also evaluated by testing non-CaPV DNA samples extracted from Orf virus and Bovine papular stomatitis virus, and cDNA derived from Peste des petits ruminants virus (Table 2). Samples were blinded to the operator and analysed in duplicate. The accuracy of the genotyping was confirmed using a previously developed dual hybridization assay [14] and capripoxvirus RPO30 gene sequencing [13].

## Results

### Assay Design and Optimization

**Figure 2:**
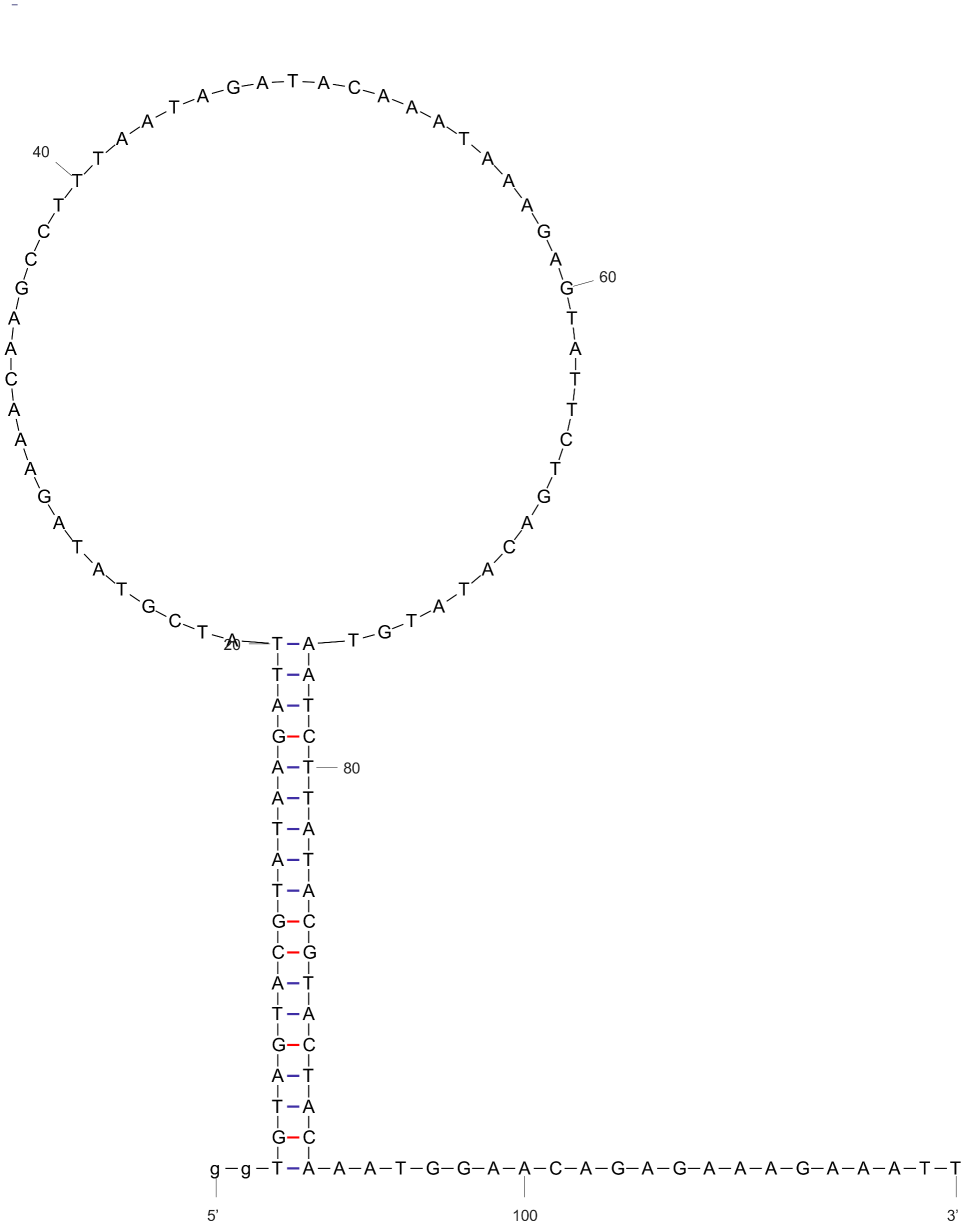
Secondary structure of the expected GTPV PCR amplicon. The Snapback hairpin with a 18-nucleotide stem and a loop of 55 bases is shown. The predictions were done on the Mfold web server [28] using the default parameters of the DNA folding form except for the temperature which was set at 45°C and the salt concentration set at 50 mM.

The assay for genotyping was designed to amplify a region of 96 bp within the CaPVs RPO30 gene. The region was selected based on the alignment of 43 CaPVs of the three species to allow their simultaneous detection and differentiation using HRM analysis of the PCR amplicons. To do so, the selected region contained 3 single-nucleotide differences between SPPV and GTPV (T:G, G:T and A:T for which the sum is only at A:T change), and four single-nucleotide changes with LSDV (T:G, G:T, A:G and A:G equivalent to 2xA:G nucleotide changes). The initial evaluation showed that LSDV could be differentiated from SPPV and GTPV, but the assay failed to differentiate GTPV from SPPV. Therefore, a snapback tail of 16 bases length was designed to match 100% with GTPV, and presented a T:A mismatch with SPPV and a T:G mismatch with LSDV, and attached to the 5′ end of the forward primer to provide a more targeted genotyping. As anticipated, the *in silico* analysis of the expected PCR amplicons showed a secondary structure with a hairpin in which, the snapback tail hybridizing on the extension product to produce a stem of 18 base pair and loop of 55 bases (Figure 2).

**Figure 3:**
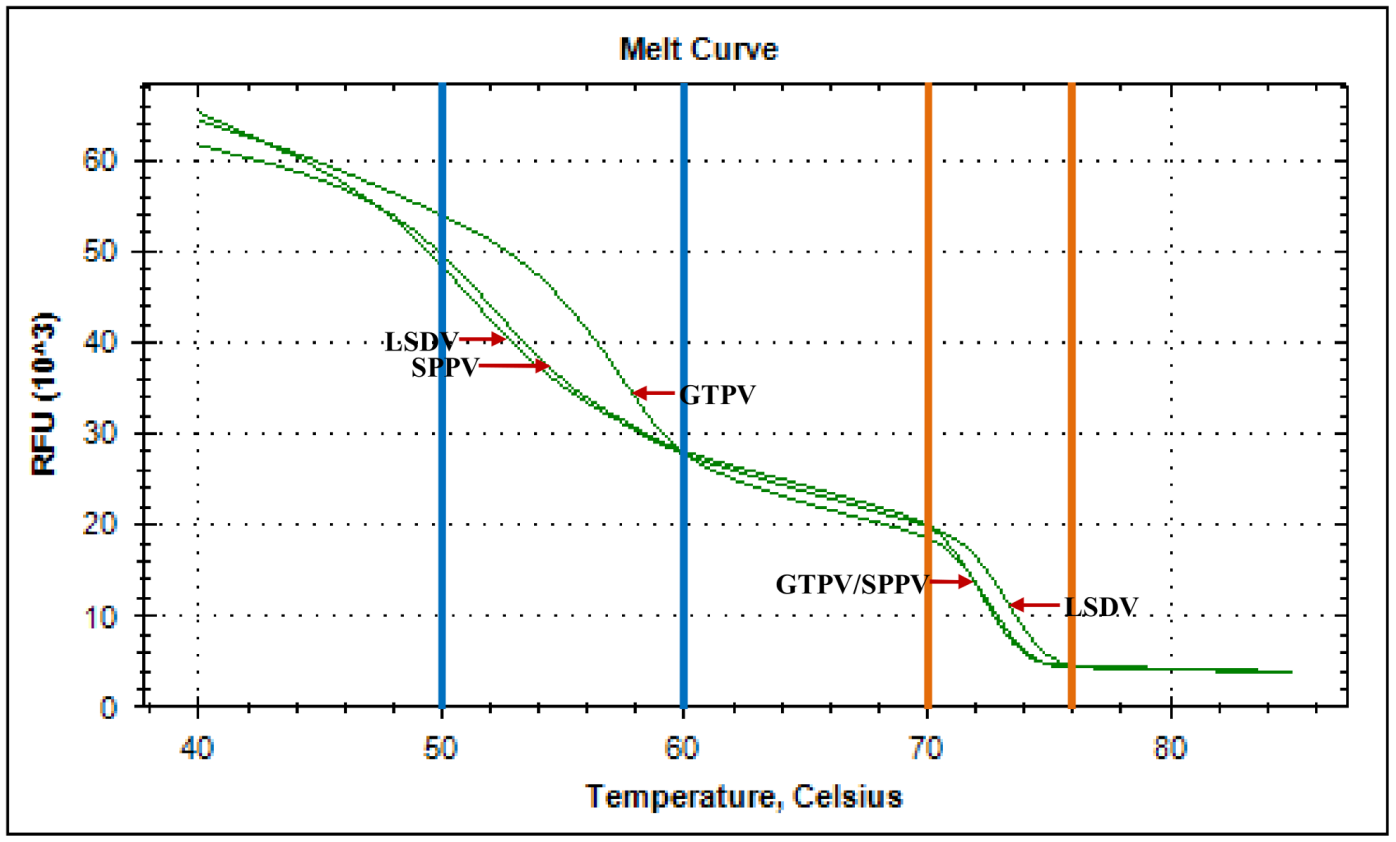
Melting curves of PCR products. Plasmids harbouring the RPO30 gene of GTPV, SPPV and LSDV were used as templates. The melting regions of the PCR products duplexes are located between the two colored thick vertical lines; the blue lines are flanking the melting region of snapback hairpins (50–60°C) while the orange lines are flanking the full-length amplicons (70–76°C).

**Figure 4:**
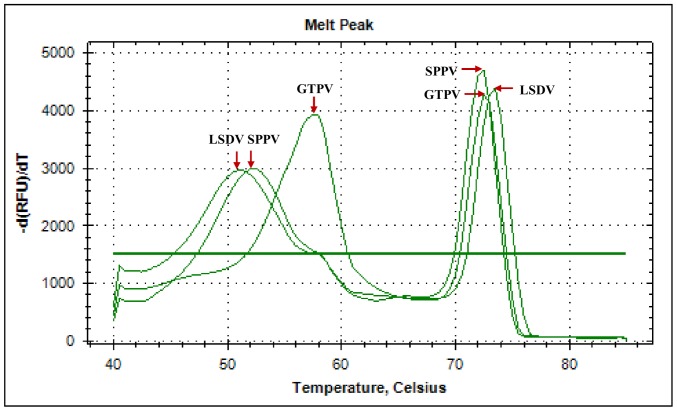
Snapback primer genotyping of CaPVs. The fluorescence melting curve analysis of the PCR products shows two melting peaks for each of the CaPV three genotypes (GTPV, SPPV and LSDV) corresponding to the snapback stem melting peak at lower temperature and the full-length PCR amplicon melting peak at higher temperature (see arrows).

With the selected primer pairs and following subsequent optimization steps, it was possible to produce an efficient amplification strategy, clear melting curve differences among the three genotypes (Figure 3), and appropriate melting peaks for both PCR amplicons and snapback stems (Figure 4). The PCR primer concentrations as well as the annealing temperature and time were found to be the most critical parameters of the assay. The optimal concentrations were 500 nM for the forward snapback primer and 40 nM for the reverse primer. Using the positive control plasmids corresponding to each of the 3 genotypes, under the optimized conditions, the following pairs of melting temperature (snapback tail, amplicons) were obtained: GTPV (58.0°C, 72.5°C), SPPV (52.0°C, 72.5°C) and LSDV (51.0°C, 73.5°C). Following a close observation and the analysis of these melting peaks, the following criteria were applied for the genotyping: the amplicons melting temperature was used to differentiate LSDV (Tm = 73.5°C) from GTPV/SPPV (Tm = 72.5°C); and the snapback melting temperature to differentiate SPPV (52.0°C) from GTPV (58°C).

### Discriminating Power of the Assay

To study the discriminating power of the assay, sixty three cell culture supernatants or clinical specimens from capripox suspected animal were tested (Table 2). Fourteen were genotyped as SPPVs, 25 as GTPVs and 24 as LSDVs. Nine of the samples collected in domestic ruminants and two samples from wildlife contained CaPVs that were outside the group corresponding to the name of their host of origin (Table2). The snapback primer genotyping results were in complete agreement with those obtained using the dual hybridization probe assay. For each of these 63 CaPV samples, the genotype was further confirmed by sequencing of the RPO30 gene, giving a sensitivity of 100%.

No amplification was observed for non-CaPV DNA from Orf virus, Bovine papular stomatitis virus, and cDNA from Peste des petits ruminants virus, even when the number of amplification cycles was increased to 50 cycles (Table 2). Furthermore, 8 additional samples from suspected capripox cases and 20 swab samples from healthy goats tested negative in the snapback assay. All these negative samples were also confirmed to be negative by dual hybridization probe assay; hence the specificity was 100%.

**Figure 5:**
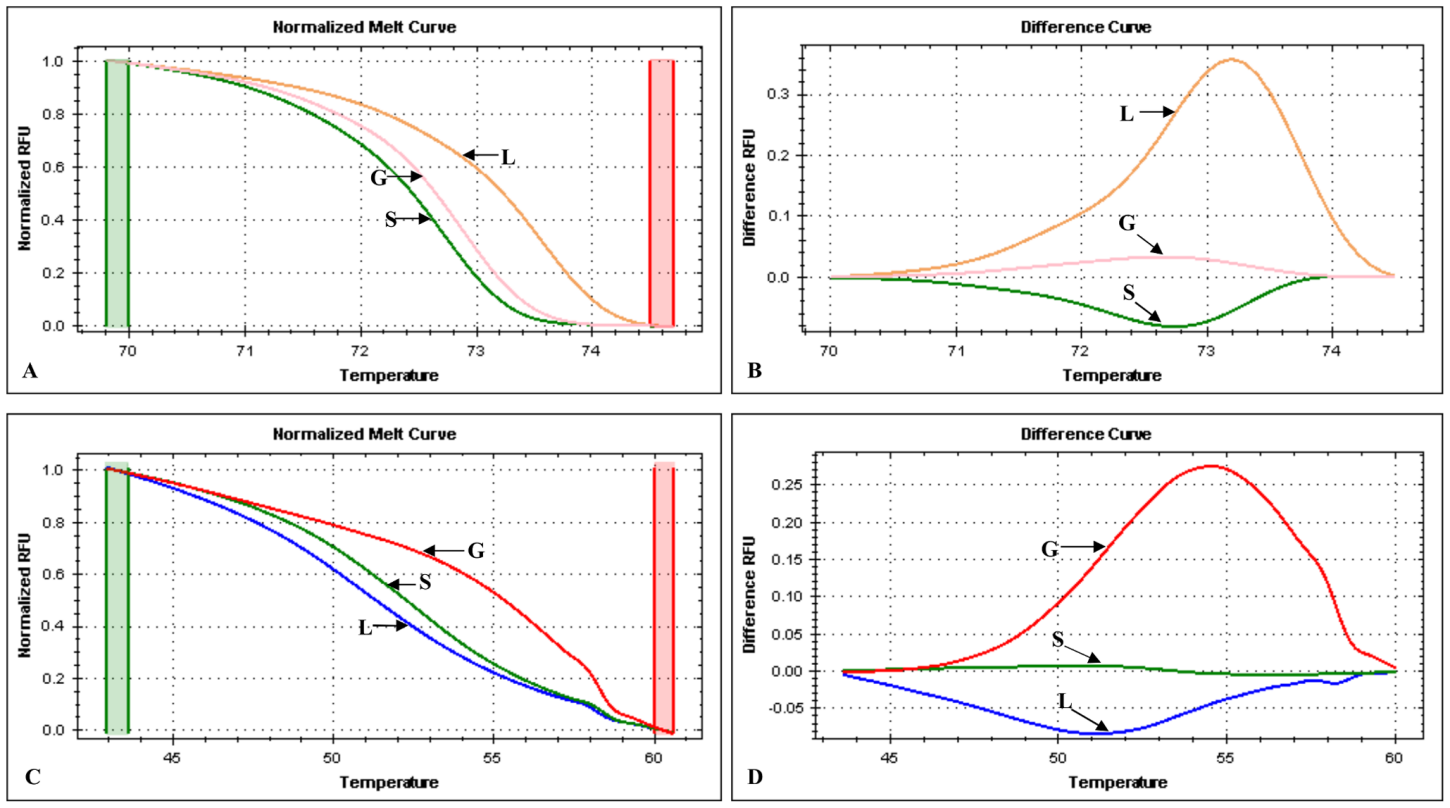
High-Resolution melting curve analysis of CaPVs using the Precision Melt Analysis™ software (BioRad). A: the normalized melt curve of the full-length amplicon; B: the difference curve of full-length amplicon; C: the normalized melt curve of snapback stem; D: The difference curve of the snapback stem. The species are indicated by the arrows: G = GTPV, S = SPPV and L = LSDV.

To further confirm the virus host origin discriminating power of the current genotyping method, the melting data of the PCR product duplexes acquired using CFX™ Manager software were analysed using the Precision Melt Analysis™ software. Since the assay produced two PCR amplicons duplexes representing the snapback hairpin and the full-length amplicons, the HRM analysis was done separately for each melting region. The normalized melt curves and difference curves were acquired by selecting the pre-and post-melt regions for the PCR amplicons duplexes by shifting on the two different melting temperature regions, thus for the full-length amplicons the data analysis window was gagged between 70°C–74.6°C and then for analysing the snapback hairpin stem the window was again adjusted between 48°C–59.5°C as shown in figure 5. The HRM analysis of the full-length amplicons revealed that LSDV was separated from GTPV and SPPV; likewise the HRM analysis of snapback hairpin resulted in the clear separation of GTPV from SPPV and LSDV isolates (Figure 5).

### Limit of Detection of the Assay

The limit of detection of each genotype was determined using probit analysis. Five replicates of different dilutions for each three genotypes were amplified on five separated occasions and the percentages of positive results were determined. The LODs at 95% confidence determined graphically were: 130 GTPV DNA copies per reaction (116–155), 168 SPPV DNA copies per reaction (54–192) and 183 LSDV DNA copies per reaction (165–211). The detection of the virus was recorded at a concentration down to 20 copies per reaction for all three genotypes, but with poor genotype determination. Genotype was correctly determined at 10 copies per reaction when amplification was increased from 45 to 50 cycles without compromising the specificity of the assay.

### Cross-platform Compatibility

To evaluate the cross-platform compatibility of the assay, we performed the assay under the conditions described in the Material and Methods section using plasmids containing each of the three CaPV genotypes in the following instruments: the 7500 Fast Real-Time PCR System (Life Technologies), the LightCycler® 480 Real-Time PCR System (Roche), the Rotor-Gene Q (Qiagen), the MiniOpticon™ Real-Time PCR Detection System (BioRad), in addition to the CFX96 ™ Real-Time PCR Detection System (BioRad).

The melting of both amplicons and snapback stems were obtained for a successful genotyping in all instruments except the 7500 Fast Real-Time PCR System, where only the melting of the amplicons could be observed. Indeed, the fixed data-acquisition capabilities (3 data points/°C) of the 7500 Fast Real-Time PCR System and the supplied version of the software (version 1.4) were not adequate for evaluating the snapback melting [24].

There was a small shift in the Tm values of both amplicons and snapback stems from one instrument to another. The following pairs of snapback tail/amplicons melting temperatures were observed: GTPV (56.50°C, 73.90°C), SPPV (52.60°C, 73.90°C) and LSDV (52.20°C, 74.90°C) on the Rotor-Gene Q (Qiagen), GTPV (56.00°C, 73.30°C), SPPV (51.40°C, 73.30°C) and LSDV (50.70°C, 74.30°C) on LightCycler 480 (Roche), and GTPV (56.00°C, 73.00°C), SPPV (51.50°C, 73.00°C) and LSDV (50.50°C, 74.00°C) with the MiniOpticon™ Real-Time PCR Detection System (BioRad).

Additionally, after performing the assay on a normal PCR machine (BioRad C1000) and transferring the amplified products to the CFX96 ™ Real-Time PCR Detection System for melting curved analysis only, we successfully detected all three genotypes.

## Discussion

A cost-effective, cross-platform compatible and easy-to-perform real time PCR assay was developed for CaPV genotyping using a snapback primer and the dsDNA intercalating EvaGreen dye. A snapback probe element added to the 5′end of the forward primer allowed the formation of a second melting peak during the melting of the PCR products, corresponding to the melting of the snapback stems. Using a combination of the melting of the snapback stems and those of the amplicons, we were able to develop a new approach for CaPV genotyping. The genotyping was achieved by using information from both snapback and amplicons melting. The melting of the amplicons was used to differentiate LSDV (Tm = 73.5°C) from GTPV/SPPV (Tm = 72.5°C), because the melting peaks separation can be more accurately determined in this region due to height of the peaks as compared to the snapback melting peaks of SPPV (52.0°C) and LSDV (51.0°C) which are more flat. Furthermore, this 1°C difference between LSDV amplicons Tm and those of GTPV/SPPV was maintained in all real time PCR machines that were used. This confirmed that the amplicons melting is the best option to differentiate LSDV from GTPV/SPPV. In contrast, the snapback stem Tm difference between LSDV and SPPV varied according to the real time PCR machine (from 1°C with the CFX and MiniOpticon of BioRad to 0.4°C with the Rotor Gene of Qiagen).

The snapback melting was used to differentiate GTPV from SPPV/LSDV due to the noticeable difference in the melting temperatures of the snapback stem of GTPV (Tm = 58°C) which matched perfectly, and those of SPPV and LSDV (Tm = 52.0°C and 51.0°C respectively) which included one mismatch each (T:A and T:G, respectively). The Tm difference of the snapback stem between GTPV and SPPV/LSDV was higher than 3.8°C in all the 4 real time PCR machines which performed successfully in the snapback genotyping.

This assay was readily able to genotype all investigated samples and assign them into one of the following three CaPV groups: SPPV, GTPV and LSDV. The results of the CaPV genotyping were in complete agreement with that of the dual hybridization probe assay [14]. As previously observed [14], we also found that some CaPVs fall outside the group corresponding to the name of their host of origin. This was the case of GTPV Saudi Arabia, and GTPV Nigeria goat vaccine, which are in reality SPPVs; SPPV Oman known to be a GTPV and SPPV KS1 which was confirmed to be an LSDV [14], [15]. Some samples collected from more recent outbreaks which were clustering outside the group corresponding to the name of their host of origin were further characterized by gene sequencing and phylogenetic reconstruction to confirm their genotypes. These were two samples from Kenya (Kitengela/O58/2011 and Kitengela/O59/2011), and three from Ethiopia (Chagni/O06/2012, Akaki/O01/2008 and Metekel/O01/2010), both collected from sheep, but identified as GTPV using our new assay. These genotyping results were in complete agreement with those of the dual hybridization probes assay [14] and the phylogenetic reconstruction using the CaPV RPO30 gene (data not shown). Additionally, two samples collected from Springbok antelope were detected as LSDVs (Table 2) which was in agreement with our previous characterization data [13] .

The snapback primer technology was first established as a closed tube genotyping method with saturating dye having a similar specificity to a probe without the need to use any covalent label [26]. It was successfully applied for the genotyping of Factor V Leiden [26], Gilbert Syndrome UGT1A1 (TA)_n_ promoter polymorphism [24] and for the enrichment and detection of rare alleles [25]. As previously reported in other assays utilizing the snapback primer strategy [24]–[26], our method was also found to be optimal using asymmetric concentrations with an excess of snapback primer and limiting the reverse primer. Despite the fact that this assay is based on an intercalating dye (EvaGreen), it remains highly specific due to the snapback strategy which increased the specificity of the test to the similar level with the fluorescently labelled probe assays.

To our knowledge, this is the first report on the application of unlabelled snapback primer strategy for pathogen genotyping. Our results show that this approach represents an effective and cheaper alternative to the fluorescently labelled probes for pathogen typing such as that described by Lamien et al. [14].

A non-labelled probe approach has been previously described for herpes simplex virus (HSV) to differentiate HSV1 from HSV2 [21]. However, at the opposite of above mentioned method, the snapback primer strategy doesn’t use any chemical modification in the unlabelled probe to prevent its extension [26]. Instead, similarly to Zhou et al. [26], we used two non-related nucleotides to block the extremity of the snapback tail of the forward primer to prevent its non-specific extension.

Although we have used an HRM curve analysis to further confirm our results, the present assay doesn’t necessarily require this analysis, since the melting peaks are sufficient for CaPV genotyping, reducing the technical difficulty for interpretation of the results.

The main weakness of this assay is its low analytical sensitivity as compared to the dual hybridization assay developed by Lamien et al. [14]. This is due to the fact that the best conditions of the amplification were not selected because an improved amplification is offset by lower snapback signal. This is probably due to the lower level of ssDNA production, and the difference in the ratio between the amplicon melting peak and that of the probe element in the snapback primer which then tend to become flat as the amplicons melting peak increases. Similar observations were made by Dames et al. [21], when working with unlabelled probes for HSV genotyping. This corroborates also the previous observations that the intra-molecular hybridization of the snapback can be favoured by lowering the nucleic acid concentration or by diluting the PCR products by 10-fold after amplification [26]. Interestingly, it was possible to genotype samples with lower copy number (down to 10 copies per reaction), without compromising the assay specificity by increasing the number of amplification cycles to 50 cycles. Nevertheless, according to our experience, the LOD values observed after 45 amplification cycles are much lower than the viral load in most the CaPVs clinical specimen.

Genotyping of CaPVs is very crucial due to the complex epidemiology picture of capripox disease. While sheep pox and goat pox are reportedly found in Africa (except southern Africa), Asia, Middle East and southern Europe [5]–[9], lumpy skin disease remains restricted to the African continent (except northern African countries) with some incursions in Egypt and Israel [4], [10]–[12].

In sub-Saharan and central African countries, it is believed that the three capripox virus species co-exist causing diseases in cattle, sheep and goats. However, CaPV nomenclature is still largely based on the host-species name, so that using currently available assays it is impossible to differentiate if the capripox disease in sheep is caused by a GTPV or a goat is affected by SPPV without genetic characterization of the virus isolate. Considering the high cost of gene sequencing, the method is not likely to be applied for routine screening and it is not affordable in most laboratories in the affected regions. It is well established that most of CaPV strains especially those affecting small ruminants are not strictly host-specific and can cross-infect both sheep and goats [13]–[15], [29]. This study does confirm this finding with five new outbreaks in sheep caused by GTPVs. The availability of a cost-effective diagnostic tool for routine determination of CaPV genotype will assist to clarify the epidemiological picture in the affected regions.

The assay presented in this paper is easy to perform and interpret. In addition, it is cheap, and thus, is likely to be affordable for veterinary laboratories with moderate resources. It is expected that the effective implementation of this assay for routine screening of capripox outbreaks will facilitate the more precise disease management and control.
